# Odontological Society of Pennsylvania

**Published:** 1897-09

**Authors:** 

**Affiliations:** Odontological Society of Pennsylvania


### ODONTOLOGICAL SOCIETY OF PENNSYLVANIA.

The regular meeting of the Society was held on February 13, 1897, the President, Dr. C. N. Peirce, in the chair.

*The President.*—We have with us this evening Dr. Henry Leffmann, who has kindly prepared a paper on "Ancient Metallurgy" for our instruction, and, if prepared, the Society will now receive it.

(For Dr. Leffmann's paper, see page 574.)

### DISCUSSION.

*Dr. Head.*—Where they poured lead on the pipe,—was it pure lead or an alloy? Did it stop the water as a plug or as a solder?

*Dr. Leffmann.*—I think it was pure lead. They probably applied it with a blow-pipe.

*Dr. Head.*—Very much in the way of the other solder you spoke of?

*Dr. Leffmann.*—They sometimes applied a solder in that way and sometimes used a blow-pipe. The skilful workmen did fairly well, but the others did very bad work. Some of it was evidently done by persons of very little experience.

*Dr. Peirce.*—Dr. Leffmann, do you suppose that the disposition on the part of the ancients to make the higher metals out of the baser was believed to be a possible thing with the intelligent, or was it simply a trick to act upon the credulity of the ignorant?

*Dr. Leffmann.*—At no time among the ancients were correct notions on the nature of metallurgy prevalent. There was a confusion of ideas. They thought that all metals were from the same source. Lead was at one time thought to be the source of the other metals. This seems singular, but we can understand it when we reflect that lead ores frequently contain gold and silver. In cupelling lead we often get notable quantities of silver and a trace of gold. The ancients thought that the gold and silver were produced from the lead. They did not realize that gold and silver were merely in the lead ore, therefore I think that the most educated persons were deceived on the subject. Even as late as 1630 the question was discussed as to why the calyces of lead and tin are heavier than the lead and tin themselves. There was confusion on this point because they did not understand that when lead oxidized it gained weight from the added oxygen.

As I mentioned in the latter part of the paper, I believe the preparation of alloys from absolutely pure metals—free from each other and from all other contaminations—will give rise to practical results. We must have some way of preparing them without oxidation, for instance, *in vacuo*. We have now positive knowledge that copper is affected by the presence of copper oxide in it. It has been long known that the presence of antimony will change the properties of gold.

There is here opportunity for painstaking research. Some of these errors of the past are prominent enough to show us how easy it is to go wrong. It was probably over-confidence that led these men astray.

*Dr. Peirce.*—Can Dr. Leffmann give us a little explanation of how it is that when we are melting metals together that tin melts at a low temperature and gold at a much higher, but putting them together they melt at a low temperature?

*Dr. Leffmann.*—The principle, I think, is both physical and chemical. In the first place, the melted tin conveys the heat to all parts of the gold. It is a close contact with the gold. Then, I think, there is a chemical combination. They enter into combination and make an alloy which is very fusible. The fusing-point of many an alloy is below that of its constituents. I think it is a sort of sugar-and-water action. The gold dissolves in the melted tin as sugar dissolves in water.

I made an experiment lately. I had often read that sodium and potassium, which are both solids, will make a liquid alloy. I tried the experiment, rubbing together a little of each. They promptly liquefied and remained so—the melting-point of the alloy being beneath that of both of the constituents, and below the temperature of the atmosphere. I think that the melting of the gold in the tin is due to a similar combination of the gold with the tin.

I once had such a combination illustrated in a peculiar way. I had some worn-out zinc plates of an electric battery, and wanted to melt the zinc plates to get the brass binding screws out. When I picked out one there was merely a shadow of it left,—the oxide and dirt. When I touched it, it fell to pieces. The brass had dissolved in the melted zinc. Now, there was a combination between the copper of the brass and the melted zinc.

In a bar of silver the copper alloy "segregates," as the assayer calls it, so that the lower part of the bar will not have the same amount as the upper part. One side of a silver dollar will sometimes assay differently from the other side, on account of the separation as the cooling of the ingot takes place.

*Dr. Head.*—When mercury dissolves metals and forms an amalgam that solidifies at the temperature of the air, would not that same phenomenon take place with tin or lead or zinc if the melting-point of those metals happened to be the normal temperature?

*Dr. Leffmann.*—I think so. The peculiarity in the case of mercury is simply due to the fact that we live at a temperature above the melting-point of mercury. If you experimented as much above the melting-point of zinc as we are above that of mercury, you would get the same series of results with zinc.

*Dr. Warren.*—I would like to ask Dr. Leffmann if the affinity between the gold and tin had something to do with the rapid melting?

*Dr. Leffmann.*—I think that the tin and gold are in different electrical states. The tin is a metal of high positive character, while the gold is a metal of low positive character, and therefore somewhat opposing conditions exist.

*Dr. Head.*—A curious chacteristic of amalgam lies in the fact that no matter how hard it sets, the mercury will leak out into another metal that is placed in contact with it. Would not that indicate a mechanical rather than a chemical combination?

*Dr. Leffmann.*—I think, perhaps, I must come back to the answer, "Both and neither." We have in most amalgams, as they are made, an excess of mercury. Now, that excess will readily go from one metal to the other. It does not follow that the combination is only a mechanical one.

I remember hearing a gentleman assert that there is lime in Portland cement, because we can wash lime out of it. That does not follow. The water may produce it from the elements present. I do not believe that the drawing out of the mercury is a proof of its presence in a free state. But I do think that there is mercury present in these amalgams in a free state.

*Dr. Warren.*—In any amalgam?

*Dr. Leffmann.*—Probably more than necessary to form the hard mass.

*Dr. Head.*—When metals are combined, does not the alloy almost always melt below the mean melting-point of all the contained metals?

*Dr. Leffmann.*—Yes. Because it is a chemical combination.

*Dr. Head.*—Why chemical?

*Dr. Leffmann.*—It is a new substance, and, therefore governed by a new law.

*Dr. Head.*—But why does it not melt at a *higher* temperature?

*Dr. Leffmann.*—I do not think we can explain that. I think we can only say that proof of chemical combination is wanting.

*Dr. Warren.*—I would suggest that the difference lies in the fact that the *cohesion* between the molecules of the pure metal is greater than the *adhesion* between the molecules of the alloy. In other words, the affinity between molecules of a like nature is greater than between those of a dissimilar nature. Is not this a fact, Dr. Leffmann?

*Dr. Leffmann.*—Yes, I think so. But here again we have a difficulty in drawing the line between cohesion and adhesion. There are different degrees. Take a solution of starch in water. You can filter a dilute solution, and you cannot see any difference. Yet if you put in diastase, it changes the starch, because the diastase gets nearer to the water.

The ancients thought there were ferments for metals,—things to put into the base metals which would transform them into the superior, so that when they put them into the mixture all would turn into gold. It is very difficult to classify these terms,—chemical combination and mechanical combination, cohesion and adhesion.

*Dr. Warren.*—In regard to the alloys in the mint, where they find more of one of the constituents on one side than on the other, is not that due to the difference in specific gravity of these metals while in the state of fusion?

*Dr. Leffmann.*—Yes, I think it is. But it is not so much the difference of specific gravity between, for instance, copper and silver as between that of an association of copper and silver and the rest of the silver. There are two combinations formed. They are indifferently miscible with each other at the melting-point, but as they cool, one mass of copper-silver alloy fails to hold the other in solution, and thus we have not copper alone, but a richer or poorer copper-silver alloy. It is the separation of one combination from the other.

The same thing is supposed to exist in glass, which has been defined to be a supersaturated solution of one silicate in another. They are solid solutions, but they have the properties of solution,

While they are solutions in the hot state, the longer they are in cooling the more perfectly do they separate.

The character of iron can be changed by having it cooled more quickly or slowly.

*Dr. Head.*—I would like to ask the doctor how it is that if lead and zinc are mixed together, as sometimes happens in our dies, the lead does not seem to form any combination with the zinc. It invariably falls to the bottom. And yet in the case of bronze, there does not seem to be that tendency for the heavier metal to go to the bottom. Is it because in one case the chemical union is stronger than in the other?

*Dr. Leffmann.*—Mr. President, these very questions are interesting as tending to show how much remains to be done in this field. It shows that there are questions constantly asked that cannot be answered from our present knowledge. We will never answer these questions aright until we discard all the present knowledge,—consider all we know as wrong and, first ascertaining the purity of everything used, proceed so as to eliminate every possible error. The error produced by the atmosphere is a very serious one. The oxides of metals are soluble in the metals themselves.

Several years ago an aluminum amalgam was put on the market. It was copper, tin, and silver. Possibly there was aluminum in it, in the sense that a small quantity of aluminum was combined with it. By placing in one grain to a hundred one might get a better alloy, although it would consist of the same things.

An English observer made some interesting observations some years ago by using a powerful press. He proved that metals would combine under great pressure without melting. He placed some pieces of marble at the bottom, which, being lighter than the metals, would have gone to the top when the metals were melted. But the marble remained at the bottom, showing that at no time had the metals been fused. They were forced together by the pressure of many tons. Here is a method of making alloys without the injurious action of heat.

*Dr. Warren.*—Has that method been used with other metals except lead and tin?

*Dr. Leffmann.*—I think not.

*Dr. Warren.*—Is that aluminum alloy to which you referred now in the market?

*Dr. Peirce.*—Yes; and there is not any more aluminum in it. I have made alloys for many years. A very favorite alloy was twelve parts of silver, ten of tin, and five of gold. It is made by melting the tin and placing the silver in in small pieces, and then the gold in small pieces. If we made it into an ingot, we found the ingot was not uniform. One side had more gold, and was harder than the other. We took the plan of having another crucible, and then poured from that right into the mould, and in that way secured it of more uniform quality throughout the ingot. It always required care to accomplish this. If that care was not exercised we had one side heavier than the other, or a different quality of material.

*Dr. Head.*—Would the doctor explain why steel gets harder when tempered?

*Dr. Leffmann.*—Steel gets larger when hardened. A steel rod that will pass easily in a ring when soft will not pass when hardened. There are several theories concerning tempering. I do not know what is the present theory among engineers. One is that the hardening causes the carbon to remain in the iron. There is no chance for the carbon, in solution, to escape. It is in the molecules of the iron, and is forced to remain. There is no perfectly demonstrated theory, however, as to the hardening of steel. There has been one suggestion, that the carbon remains because the steel is chilled so quickly that it does not have a chance to escape. By chilling it quickly it is actually forced to remain in its place.

*Dr. Head.*—That is a very interesting theory; and still more interesting in the light of the fact that if one should take a small steel wire and heat to a cherry-red, when it cools in the atmosphere, it will not get soft, the atmosphere cooling it so quickly that it still has some temper; while if that wire is heated to a cherry-red and then plunged into water, the moment it loses its red it is found to be soft. An old mechanic taught me this years ago. This fact is certainly incompatible with the theory spoken of by Dr. Leffmann.

INCIDENTS OF PRACTICE.

*Dr. Peirce.*—I have been using a solution of alum to prevent the accumulation of tartar; over a dozen patients have tried it, and I have been surprised at the excellent results. I tell them to take a glass of water with a pinch of alum in it, and rinse the mouth freely once a day. It is harmless to the teeth, and has kept the gums in good condition, where previously there was a heavy accumulation every month or six weeks. I wish the gentlemen present would experiment and report.

*Dr. Head.*—Have you used it in conjunction with pyorrhœa?

*Dr. Peirce.*—No; but it removes the accumulation of tartar, so there would be that much irritation removed.

*Dr. Head.*—Dr. Leffmann, perhaps you could tell us whether you would consider alum harmful to the tooth-structure?

*Dr. Leffmann.*—I do not think alum would produce any corrosion. Alum is not an active corrosive agent. I should therefore not expect much corrosion from the alum. If the quantity were large, it might have an astringent action on the gums. I feel that the experiment would probably show very little, if any, corrosion of the teeth proper. Alum is not like a free acid. It has the properties of an acid, but simply because there is a want of balance between the alumina and sulphuric acid. It shows the properties of an acid to litmus-paper and to our taste. But there is as much neutralizing material in it as in baking soda. We are apt to think that alum is an acid substance, when in reality it is merely a substance with an acid reaction.

*Dr. Broomell.*—Under the head of incidents of practice I have a case that I feel like reporting to the Society. It is a case of what might be called "bite interference." It is in the mouth of a young lady, twenty-six years of age. She has been under my care for at least twelve years, and I have suddenly noticed a change in her articulation,—such a change that the anterior teeth do not occlude by an eighth of an inch. Previously there was an over-bite of an eighth of an inch, the superior teeth overhanging the inferior. I examined the wisdom-teeth to see if it was the result of their entering the arch. That is not the cause. There is probably some change in the inferior maxilla itself. I would like to ask the members for an opinion as to the cause and the proper treatment of the case.

*The President.*—You do not think that the anterior teeth have been shortened by being thrown forward?

*Dr. Head.*—I judge we have all had patients who are in the ultimate stage Dr. Broomell speaks of. But I, for one, have not had the good fortune to see the process under development. Heretofore I thought that when the back teeth struck and the front teeth failed to join by an eighth of an inch or more, it was due to an excessive growth of the alveolus in the bicuspids or a lack of growth in the alveolus of the centrals. But from the cases I now remember, and there are three in my own practice, it would seem that it was from an excessive growth in the molars. I would be much pleased if a remedy could be found.

*Dr. Broomell.*—I cannot understand, Mr. President, how an excess of growth in the alveolus could effect this; because it is natural for the teeth to have opponents. I cannot understand it, unless there is some change in the angle of the jaw. I have accused the young lady of a habit of extending her chin, resulting in a change in the convoloid process.

*Dr. Head.*—Still, it might also be due to the fact that this particular patient, while keeping the teeth apart, might give them a chance to come down, even in a short period. The difficult part to explain is why the front teeth should not do it as well as the back.

Adjourned.

JOSEPH HEAD, M.D., D.D.S.,
*Editor Odontological Society of Pennsylvania.*

